# Pre-Launch Absolute Calibration of CCD/CBERS-2B Sensor

**DOI:** 10.3390/s8106557

**Published:** 2008-10-23

**Authors:** Flávio Jorge Ponzoni, Bráulio Fonseca Carneiro Albuquerque

**Affiliations:** 1 Instituto Nacional de Pesquisas Espaciais, Divisão de Sensoriamento Remoto, Avenida dos Astronautas 1758, 12227-010, São José dos Campos, SP, Brazil; 2 Instituto Nacional de Pesquisas Espaciais, Divisão de Engenharia Aeroespacial, Avenida dos Astronautas 1758, 12227-010, São José dos Campos, SP, Brazil. E-Mail: braulio@dea.inpe.br

**Keywords:** Absolute calibration, CCD/CBERS-2B sensor, pre-launch calibration

## Abstract

Pre-launch absolute calibration coefficients for the CCD/CBERS-2B sensor have been calculated from radiometric measurements performed in a satellite integration and test hall in the Chinese Academy of Space Technology (CAST) headquarters, located in Beijing, China. An illuminated integrating sphere was positioned in the test hall facilities to allow the CCD/CBERS-2B imagery of the entire sphere aperture. Calibration images were recorded and a relative calibration procedure adopted exclusively in Brazil was applied to equalize the detectors responses. Averages of digital numbers (DN) from these images were determined and correlated to their respective radiance levels in order to calculate the absolute calibration coefficients. It has been the first time these pre-launch absolute calibration coefficients have been calculated considering the Brazilian image processing criteria. Now it will be possible to compare them to those that will be calculated from vicarious calibration campaigns. This comparison will permit the CCD/CBERS-2B monitoring and the frequently data updating to the user community.

## 1. Introduction

Since 1988 China and Brazil have carried out a joint space program called China-Brazil Earth Resources Satellite (CBERS), specially dedicated to environmental data assessment. In the beginning, the CBERS program included two remote sensing satellites (named CBERS-1 and CBERS-2) with three different sensors onboard: the Wide Field Imager (WFI), the High Resolution CCD Camera (CCD) and the Infrared Multispectral Scanner (IRMSS). CBERS-1 and CBERS-2 were launched in October, 1999 and November, 2003, respectively. The relative success of these two satellites in both engineering and application terms encouraged Chinese and Brazilian governments to expand the cooperation and to include five new satellites in the CBERS program: CBERS-2B (2007), CBERS-3 (2009), CBERS-4 (2011), CBERS-5 (2013) and CBERS-6 (2015).

Considering the four years between the end of CBERS-2 lifetime (2005) and CBERS-3 launching (2009) it was decided to launch the CBERS-2B satellite in order to guarantee the continuity of several environmental monitoring programs in both countries.

CBERS-2B payload is composed by the CCD, the WFI and by a new camera, named High Resolution Camera (HRC), flying for the first time onboard a CBERS satellite. In spite of the expected new possibilities to be explored with this improvement in nominal spatial resolution (2.36 m) aspect, main attention is still expected to the CCD data quality, since at least among the Brazilian remote sensing users, there are a lot of researches (academia) and application initiatives (government and private companies) that depend on its data. Some of these research projects and general applications have been based on the conversion of digital numbers (DN) to physical unities like radiance or reflectance.

Taking into account well succeeded remote sensing programs in the world, all of them have been characterized by a continuous concerning to properly inform the users about the relationship between DN values and the effective in-flight radiance (Top of the Atmosphere – TOA radiance) measured by the sensor [1]. So, it is obvious that the CBERS program success is also dependent on the same strategy.

The free CBERS-2 data access policy that has been adopted by the National Institute for Space Research (INPE) in Brazil and due differences in the image production (mainly in relative calibration) criteria adopted by the two countries (China and Brazil), encouraged Brazilian researchers and engineers to performed their own in-flight absolute calibration campaigns [2, 3] and closely participate of pre-launch absolute calibration tasks performed in China.

The objective of this paper is to describe the CCD/CBERS-2B pre-launch absolute calibration coefficients calculations based on data collected in the satellite integration and test hall located in China and relatively calibrated images generated in Brazil.

## 2. CCD/CBERS-2B sensor short description

The CCD/CBERS-2B sensor has the same technical characteristics of its predecessor ones onboard of CBERS-1 and CBERS-2 satellites. It provides images with 113 km of swath with 20 m spatial resolution and also has a lateral pointing capability of ± 32°. It operates in 5 spectral bands such as: CCD1 (0.45 – 0.52 μm -blue); CCD2 (0.52 – 0.59 μm - green); CCD3 (0.63 – 0.69 μm - red); CCD4 (0.77 – 0.89 μm - near infrared) and CCD5 (0.51 – 0.73 μm – panchromatic). A complete coverage cycle of the CCD camera takes 26 days.

**Figure 1.**
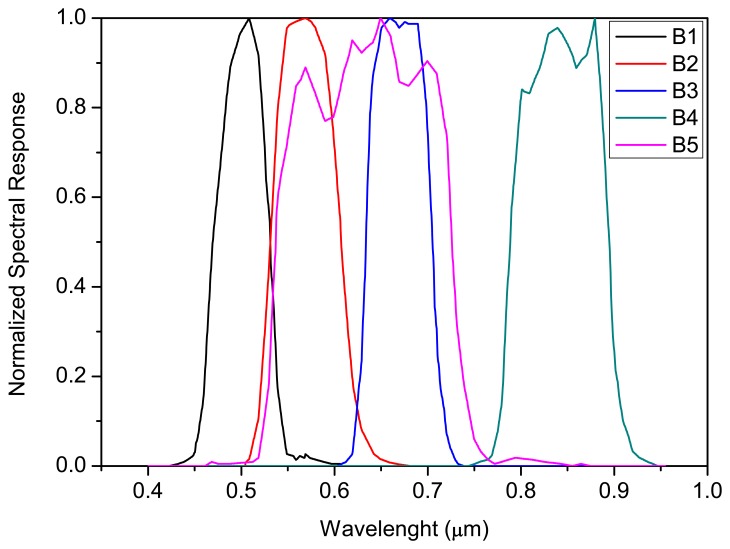
CCD/CBERS-2B normalized spectral response.

Figure 1 shows the actual spectral response of the CCD/CBERS-2B bands. These curves were originated from spectral measurements performed in laboratory taking into account the response per radiance unit. In Figure 1 the data have been normalized (from 0 to 1) to facilitate relative comparison between bands.

Basically the CCD/CBERS-2B is composed by four pieces of equipment: the Optical Mechanism, the Optical Mechanism Control Box, the Thermal Controller and the Camera Electronics. In calibration terms, the most important equipments are the Optical Mechanism and the Camera Electronics. Here, emphasis will be dedicated to the Camera Electronics equipment. Basically, this equipment takes the CCD signal generated in each band and converts them into video output signals through two channels in which the gain control is made. According to this equipment conception, signals from bands 1, 3 and 5 pass through channel 1, while signals from bands 2, 3 (again) and 4 pass through channel 2.

The redundancy conception has also been applied at this equipment. Thus it is possible generating video output signals considering “main” and “redundant” options that must be considered in the absolute calibration coefficients calculations.

The video output signals from the Camera Electronics equipment go toward an independent satellite sub-system called CCD_DT, in which there is equipment called Encoder that converts the video input signals into 8 bits digital data. The Encoder also runs with redundancy. Thus, the absolute calibration coefficients calculations must consider four equipment combinations: main (Camera Electronics) – main (Encoder), main (Camera Electronics) – redundant (Encoder), redundant (Camera Electronics) – main (Encoder) and redundant (Camera Electronics) – redundant (Encoder).

The adoption of any of these combinations influences the relationship between the effective measured radiance at the top of the atmosphere and the resulting Digital Numbers (DN), i.e. it influences the CCD/CBERS-2B absolute calibration.

It is possible to change the sensor gain to values of 0.59, 1 and 1.69. So, the calibration coefficients calculations were performed for each of these gain values. Finally, as the band 3 signals can be transmitted by two channels, specific calibrations coefficients were also determined for these two options as well.

## 3. Relative calibration

**Figure 2.**
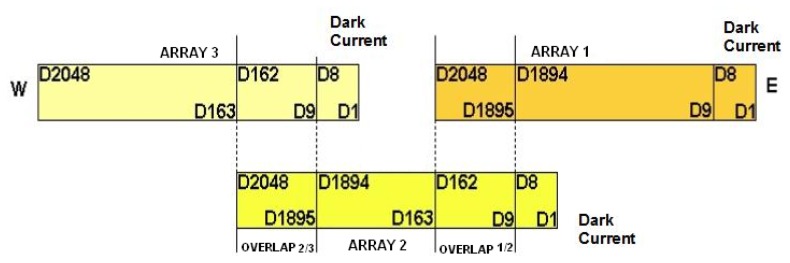
Structure of the CCD/CBERS-2B images.

The CCD/CBERS-2B raw images in each band are composed by five parts (three arrays and two overlap regions) as presented on Figure 2.

According to Figure 2, the CCD/CBERS-2B detector array arrangement consists of three arrays of 2,048 detectors (or pixels) with two overlap regions of 154 detectors and a dark current region of 8 detectors in each array. These arrays are positioned inside the camera as a “divoli” [4] conception guaranteeing the complete and continuous cross-track imagery. This conception is the same adopted in the Haute Resolution Visible sensor (HRV) on board of the SPOT satellites.

A relative calibration procedure is applied to equalize the detectors response inter and intra arrays. The first step of the relative calibration is the odd/even detectors equalization assuming that the intra-array detectors should have the same average value. This procedure is applied in each array individually, followed by inter-array equalization using the average of each array by the mean of the three arrays as a normalizing criterion. In both steps, gains and off-sets are calculated per detectors and spectral band.

In the overlap regions, as they are characterized by a low energy incidence, also two steps are followed. The first one includes the equalization intra-overlap region based on the weighted mean (by the relative distance) between homologous detectors responses and the second step includes a gain and off-set calculation to be applied in the equalized overlap region to also equalize that with two neighbors arrays responses.

The relative calibration criteria have been defined differently in China and in Brazil, since Chinese and Brazilian teams have worked independently due to the lack of a formal and specific agreement. The procedures briefly described above have been adopted by the Brazilian team according its own background and previous experience. Thus, it is expected radiometric differences between relative calibrated images generated in both countries, i.e., for the same raw data from the same scene, the resulting relative calibrated images will present light differences between digital numbers (DN) from the same pixel. These differences have not been linear and frequently they have been expressed by 1 to 20 DN in each band.

## 4. Absolute calibration

The radiometric measurements were performed in a specific integrating and test hall of the Chinese Academy of Space Technology (CAST 529 hall) headquarters located in Beijing from May 8-9, 2007.

During the radiometric measurements the CBERS-2B satellite was positioned upward (inverse flight position) near a large internally illuminated integrating sphere. A 45° inclined mirror was positioned on the CCD/CBRS-2B optic system in order to collect the electromagnetic radiation reflected from the integrating sphere internal surface.

The absolute calibration procedure took into account all possible mentioned equipment combinations (main-main, main-redundant, redundant-main and redundant-redundant), thus absolute calibration coefficients have been calculated for each of these combinations.

**Figure 3.**
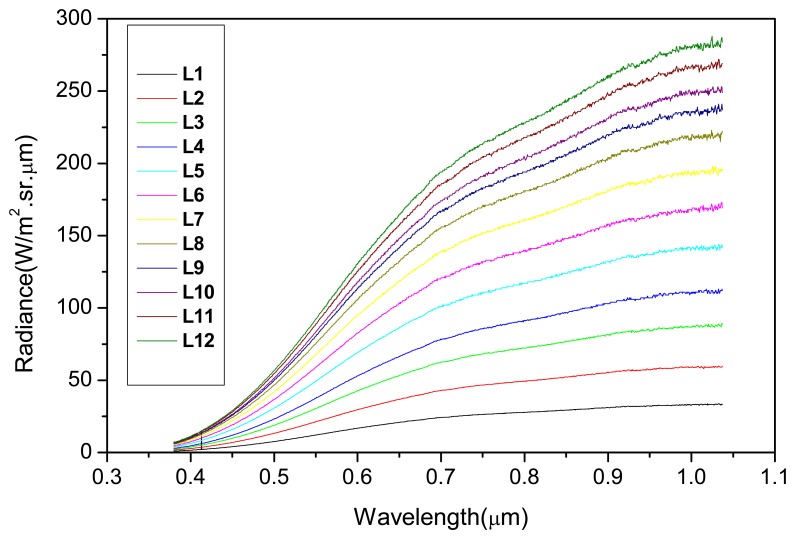
Integrating sphere radiances (L_λ_) for each illumination level considered.

Just before the CCD/CBERS-2B absolute calibration procedure, the integrating sphere characterization was performed using a calibrated radiometer (running from 380 to 1050 nm) positioned in a way where the radiance was measured through the same 45° inclined mirror. Twelve illumination levels (radiance levels) from the integrating sphere were considered in its characterization as well as in the CCD/CBERS-2B calibration. Figure 3 shows the spectral radiance (L_λ_) values for each illumination level measured with the radiometer. These illumination levels were defined taking into account the non-saturation ranges per spectral band in order to guarantee the linear relationship between radiance (L_λ_) and DN values.

The second step was the determination of the spectral radiances effectively measured by the CCD/CBERS-2B camera (Lccd_i_) in each of its spectral band. The calculation was performed with equation 1:

(1) $${\text{Lccd}}_{i}=\frac{\int_{0}^{\infty }L_{\lambda }.S_{i\lambda }.d_{\lambda }}{\int_{0}^{\infty }S_{i\lambda }.d_{\lambda }}$$

where: Lccd_i_ is the average spectral radiance (W/m^2^.sr.μm) across the CCD/CBERS-2B camera bandpass i for the particular integrating sphere illumination level; L_λ_ is the spectral radiance (W/m^2^.sr.μm) of the integrating sphere at wavelength λ, and S_iλ_ is the relative spectral response at wavelength λ in CCD/CBERS-2B spectral band i. (see Figure 1)

At the end of the radiometric measurements raw images from the integrating sphere (12 illumination levels × 5 spectral bands × 4 equipment combinations × 3 gain values) were available. An additional 12 illumination levels × 3 gains × CCD 3 optional transmission channel DN values were also available.

Depending upon the equipment combination, the spectral band and the illumination level, some saturated images occurred, which were discarded and not considered in the absolute calibration coefficient calculations.

## 5. Absolute calibration coefficients calculations

**Figure 4.**
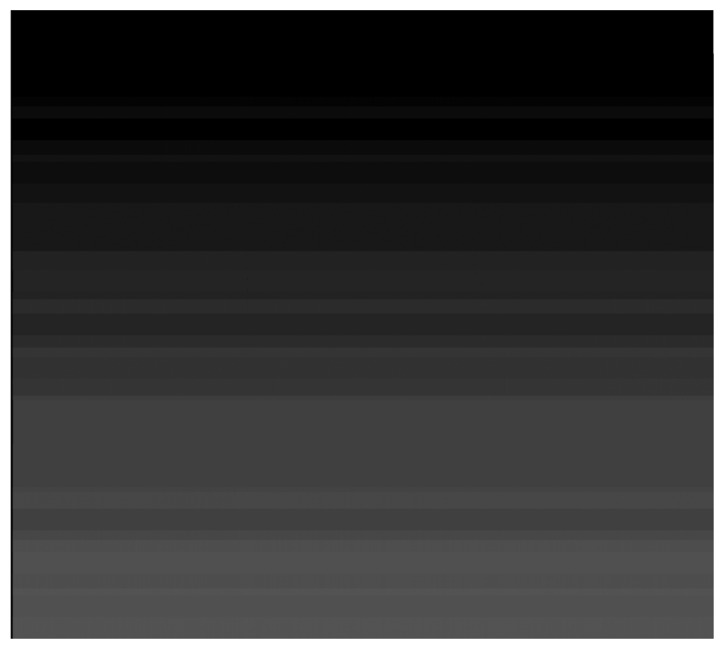
Example of a calibration image (after the relative calibration algorithm application) for main-main sub-system combination, CCD2 and gain 1. The horizontal strips represent the illumination levels.

The radiometric measurements performed in-lab conditions were materialized as the so called “calibration images”, whose DN were associated with the twelve illumination levels from the integrating sphere (from Lccd_i_). The relative calibration procedure described above (that has been adopted in Brazil) was applied to these calibration images from which the resulting DN values were extracted to calculate the absolute coefficients. Figure 4 shows an example of a calibration image.

It was calculated averages from the DN values per each illumination level that were associated with the integrating sphere radiances (Lccd_i_) in W/(m^2^.sr.μm). In this DN average calculations it was not included the “dark pixels” neither those located in the overlap region (between arrays). The dark current was also determined through the generation of images with no illumination (“dark images”). Table 1 presents the radiance values for each band and for each specific illumination level.

**Figure 5.**
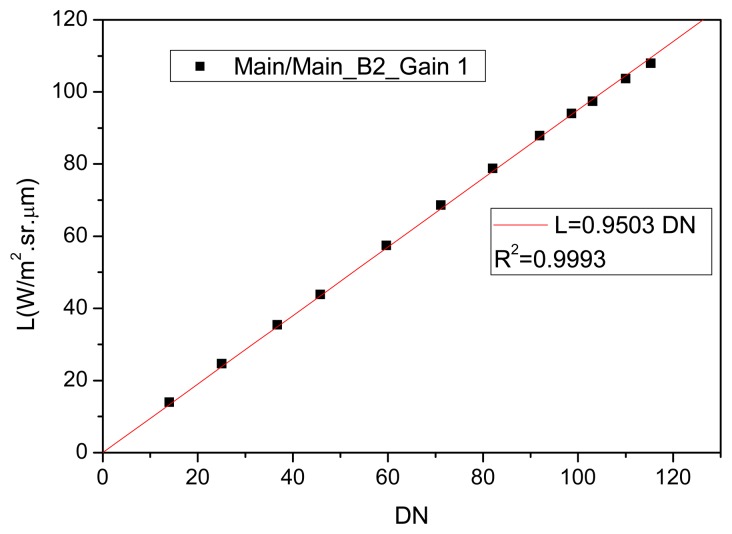
Example of the absolute calibration coefficient determination for main-main sub-system combination, CCD2 and gain 1.

The absolute calibration coefficients were determined by the relation Lccd_i_/DN_i_, where “i” represents the CCD/CBERS-2B spectral band. Figure 5 shows an example of the absolute calibration coefficient determination considering the Figure 4 data (main-main sub-system combination, CCD2 and gain 1).

Observing Figure 5, the radiometric response at main-main sub-system combination, CCD2 and gain 1, was linear and as the interception of the linear regression was forced to “zero”, the calibration coefficient was represented by the slope of the straight line. The absence of offset values in the calibration coefficients is explained by the discount of the dark current during the image processing procedure. This criterion was adopted for all the Lccd_i_/DN_i_ relationships.

As the instrument was designed to have its saturation in the AD converter and not in the CCD detector, non-linearities in the camera response to the spectral radiance have not been observed in any case. Illumination levels that caused saturation in the instrument were not used in the linear regression modeling.

Table 2 presents the absolute calibration coefficients for all possible equipment combinations, gains, transmission channels and spectral bands.

The spectral radiance calculated with these coefficients has standard deviation value less than 10%, as showed by the error budget performed that includes the traceable spectral radiance standard source error, the error in the spectrometer calibration using this traceable source, the error in the measurement of the integration sphere radiance, also considering its instability, and the transfer error of spectral radiance from the integration sphere to the CCD camera.

The application of specific coefficients depends on the gain and on the equipment combinations adopted during the imagery. The absolute calibration coefficients presented on Table 2 can be applied exclusively on the Brazilian images.

The information about gain and equipment combinations can be acquired from the image distributor, in this case, the National Institute for Space Research (INPE), directly from the image catalog available at the internet (http://www.dgi.inpe.br/CDSR/).

As a result of these calibration coefficients application one can obtain the so called TOA radiance or apparent Bidirectional Reflectance Factor (BRFapa) images whose data can be related to geophysical or biophysical parameters from different targets in the Earth surface.

## 6. Conclusions

The absolute calibration coefficients presented here have not been in-flight validated. A full and deep investigation must be carried out by the Brazilian user community. This validation must include the spectral characterization of different targets, which only will be achieved by the adaptation of atmospheric correction codes for the CCD/CBERS-2B sensor configuration.

It is also necessary to carry out vicarious calibration campaigns to monitor the coefficients dynamic and to maintain updated the vital information for trusted radiometric conversion of the DN to physical values. Vicarious calibration campaigns have been planned to be carry out in Brazil, China and United States territories taking into account the Committee on Earth Observation satellites (CEOS) orientations (www.earthobservations.org) that have been defined in the CEOS implementation plan for space-based observations for Global Earth Observation System of Systems (GEOSS). The idea is to adequate the absolute calibration procedures of the Brazilian environmental orbital sensors in the GEOSS/CEOS criteria to guarantee trusted DN to physical values conversion.

## Figures and Tables

**Table 1.** Radiance (Lccd_i_) values in W/(m^2^.sr.μm) that were associated to the homologous DN values.

**Illumination** **level**	**L_is_ CCD1** **W/(m^2^.sr.μm)**	**L_is_ CCD2** **W/(m^2^.sr.μm)**	**L_is_CCD3** **W/(m^2^.sr.μm)**	**L_is_ CCD4** **W/(m^2^.sr.μm)**	**L_is_ CCD5** **W/(m^2^.sr.μm)**
**1**	7.7897	14.0294	22.1857	29.1455	19.2294
**2**	13.6293	24.6644	39.2032	51.8179	33.9314
**3**	19.3980	35.4301	56.9003	76.1078	49.1216
**4**	23.8207	43.8673	71.0530	96.0957	61.2218
**5**	31.5034	57.4581	92.1529	123.1867	79.5860
**6**	37.6213	68.5937	109.9660	146.7388	94.9780
**7**	43.1250	78.7907	126.4603	169.0492	109.1940
**8**	47.9975	87.8769	141.4820	189.8348	122.0724
**9**	51.2125	94.0010	151.7561	204.4927	130.8632
**10**	52.8633	97.4455	158.2137	214.8800	136.2423
**11**	56.1243	103.6829	168.6138	229.6834	145.1387
**12**	58.2665	107.9275	176.2143	241.0791	151.5292

**Table 2.** Absolute calibration coefficients for all possible equipment combinations, gains, transmission channels and spectral bands.

**Gain 0.59**	**CCD1**	**CCD2**	**CCD3_1**	**CCD3_2**	**CCD4**	**CCD5**
**MM**	1.7135	1.6224	1.5881	1.6267	1.336	2.22
**MR**	1.3897	1.3142	1.2962	1.3250	1.0737	1.8017
**RM**	1.6861	1.6016	1.7588	1.6071	1.324	2.1833
**RR**	1.3531	1.2968	1.278	1.2971	1.0638	1.7643
**Gain 1.00**	**CCD1**	**CCD2**	**CCD3_1**	**CCD3_2**	**CCD4**	**CCD5**

**MM**	0.9996	0.9503	0.9376	0.9605	0.7827	1.2986
**MR**	0.8098	0.7678	1.0268	0.7852	0.6296	1.0551
**RM**	0.9875	0.9522	0.9407	0.9487	0.7823	1.2779
**RR**	0.7919	0.7655	1.0201	0.7685	0.6298	1.0321
**Gain 1.69**	**CCD1**	**CCD2**	**CCD3_1**	**CCD3_2**	**CCD4**	**CCD5**

**MM**	0.5854	0.5782	0.5763	0.5762	0.4777	0.7693
**MR**	0.4718	0.4733	0.4686	0.4687	0.3851	0.6256
**RM**	0.5868	0.5626	0.5645	0.5808	0.3851	0.7757
**RR**	0.4738	0.4589	0.4557	0.4687	0.4666	0.6256
